# Design and Evaluation of an Online Squat Fitness System: Lessons Learned During the Early COVID-19 Pandemic in Japan

**DOI:** 10.3389/fdgth.2021.679630

**Published:** 2021-05-28

**Authors:** Tianyi Wang, Masamitsu Kamon, Shima Okada, Shuji Sawada, Rui Ogawa, Naruhiro Shiozawa, Shuichi Machida

**Affiliations:** ^1^Department of Robotics, Faculty of Science and Engineering, Ritsumeikan University, Kusatsu, Japan; ^2^Ritsumeikan Global Innovation Research Organization, Ritsumeikan University, Kusatsu, Japan; ^3^Center of Innovation (COI) Project Center, Juntendo University, Tokyo, Japan; ^4^Center of Innovation (COI) Site, Tokyo University of the Arts, Tokyo, Japan; ^5^Faculty of Sport and Health Science, Ritsumeikan University, Kusatsu, Japan; ^6^Graduate School of Health and Sports Science, Juntendo University, Inzai, Japan

**Keywords:** COVID-19 pandemic, lessons learned, online fitness, physical activity, squat

## Abstract

COVID-19 has changed our lives and limited our ability to have adequate physical activity (PA). It is necessary to replace outdoor PA with home-based fitness. However, people lack access, skills, and even motivation for home-based fitness. To address these issues, we designed a free access self-monitoring and coaching and music-based interactive online squat fitness system. Body weight squat was utilized for fitness exercise and evaluated based on three indices: knee width, hip depth, and rhythm. An online survey on changes in exercise due to the COVID-19 pandemic and exercise habits was conducted to investigate the effect of the COVID-19 pandemic on PA. We collected data from 557 respondents 5 months after the system first released and analyzed 200 visitors' performance on squat exercise and the other relevant parameters. Visitors were divided into three groups according to their age: younger, middle, and older groups. Results showed that the younger group had better squat performance than the middle and older groups in terms of hip depth and rhythm. We highlighted the lessons learned about the system design, fitness performance evaluation, and social aspects, for future study of the design and development of similar home-based fitness systems. We provided first-hand results on the relation between the COVID-19 pandemic and physical exercise among different age groups in Japan, which was valuable for policy making in the post-COVID-19 era.

## 1. Introduction

On April 7, 2020, which was the beginning of a new fiscal and school year in Japan, the local government declared a state of emergency in response to the COVID-19 pandemic for Tokyo, Osaka, and five other prefectures (1). After 1 week, the state of emergency was extended to all prefectures from April 16 (2). Schools and universities were closed, and cultural and sports events were canceled or delayed. Students were asked to take lessons online, and company employees were asked to work from home.

Staying at home was necessary to curb the spread of the COVID-19, but it may lead to a lot of stress, anxiety (3), mental health problems (4, 5), and negatively affect sleep pattern (6). Moreover, spending more time at home to keep distance may limit citizens' ability to engage in physical activity (PA) to maintain health and reduce the risk of obesity, diabetes, or other chronic diseases. Recent research showed that sedentary people increased and worldwide PA decreased rapidly during the COVID-19 pandemic. In Japan, from February 11 to June 1, the daily step count decreased up to 30% (7). An online survey demonstrated a significant decrease in PA time in April, when compared to January 2020 in older adults (8).

The best way to overcome this problem is to replace outdoor activities with home-based activities. It is recommended that exercises using nothing but one's own weight, such as squat, shoulder press, and sit up, are optimal choices for indoor exercise, especially for the elderly or less well-trained people during COVID-19 (9). The use of home-based fitness has increased, and with the current norms of strict social distance and home confinement rules, exercising at home seems to be the new normal and lifestyle for the foreseeable future (10, 11). However, insufficient information about exercise programs, lack of proper training skills, and long-term motivation for online fitness have become other difficulties that we are facing now (12). Development of and access to a free online fitness system through non-profit and government entities is expected (13).

Previous studies have reported significant decrease in physical activity (8, 14). Visser et al. underlined that negative impact of the COVID-19 pandemic on PA may increase the risk of frailty, sarcopenia and disability (15). It was noticed that most studies that focused on the relation between COVID-19 and PA used qualitative approach, such as online survey and questionnaire. To our best knowledge, there is still no quantitative evaluation about the effect of decreased PA on physical performance. Moreover, insufficient information and knowledge about practicability of online fitness system left another open question: what should we notice when considering developing an online fitness or healthcare system in response to the COVID-19 pandemic?

As a consequence, the first purpose of this study is to introduce a new online fitness system: Biosignal Art, which is an open access, includes self-monitoring and coaching, as well as music, and is an interactive online fitness system. The second purpose is to evaluate the developed system by combining exercise performance and the results of exercise change during the early COVID-19 pandemic. We believe that the lessons learned from our study, with respect to either the system design or the system setup, will clearly benefit any future study with similar objectives. More importantly, considering the potential for increasing the risk of health, knowledge about PA performance in different populations during the early COVID-19 pandemic could contribute valuable information for government policy and effort for the next period of the COVID-19 era.

This paper consists of six parts. Section 2 describes the design of the proposed fitness system. The system evaluation is presented in section 3. Section 4 presents the results. Discussions and lessons learned from this study are elaborated in section 5. Finally, the conclusions of this study and future direction are presented in section 6.

## 2. System Design

### 2.1. Structure and Interface

**Figure 1:**
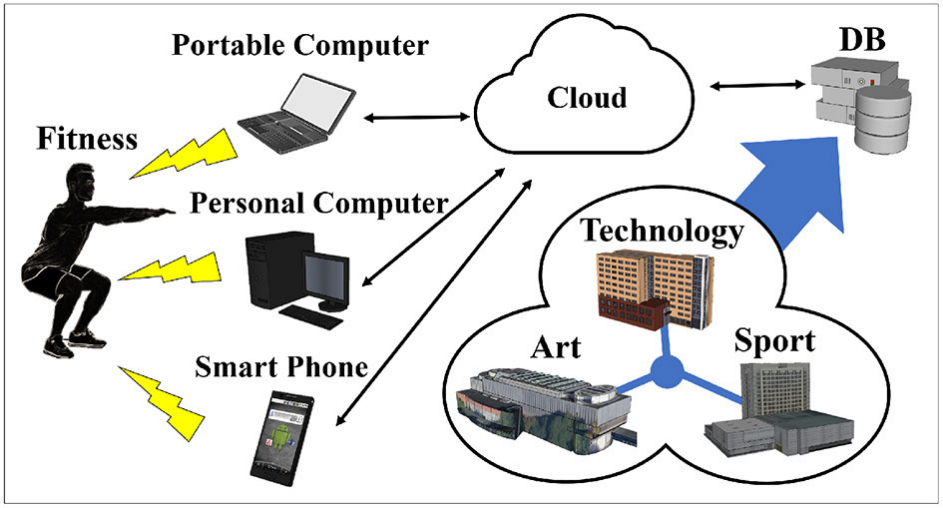
Overview of system structure.

Figure 1 shows the structure of the developed system. This system can be used on a portable and personal computer with a camera for Windows (Chrome browser) and Mac (Chrome and Safari browser) systems. Visitors can only use Android phones to access the system, at present. The Google Cloud platform was used for uploading and saving data. No collected data are linked to personal identifiable information. Video can only be downloaded by the visitors.

**Figure 2:**
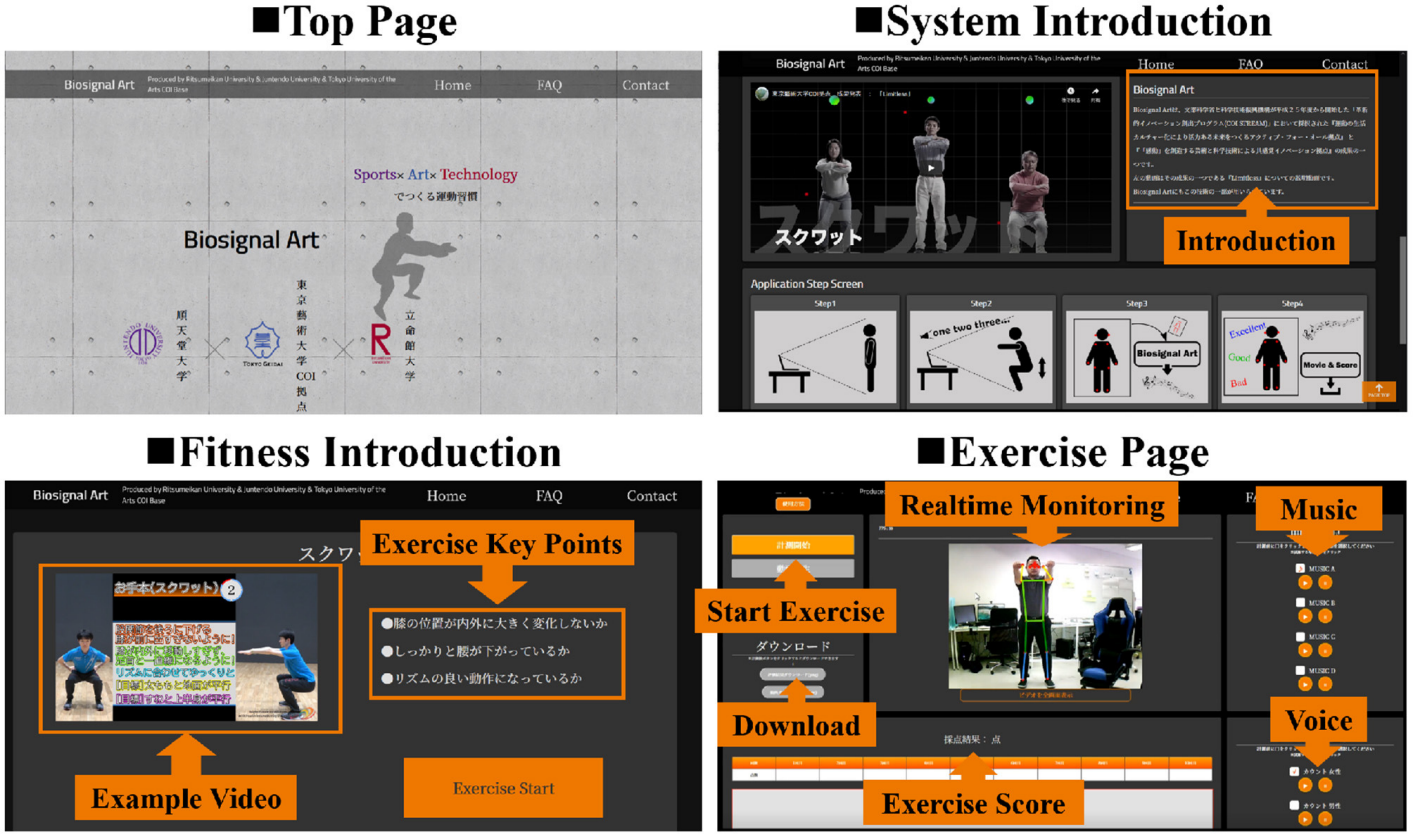
Samples of system interface.

Figure 2 shows examples of the system interface. Currently, the system interface language is in Japanese. We designed both literal and video introductions to inform the concept of our system. For the fitness introduction, we provided an example video to explain and coach visitors to perform the right exercise. Some exercise key points, which were utilized as performance evaluation indices, are also introduced. After visitors agreed to the privacy policy and terms of service, they could enter the exercise page. Visitors could choose music from four options, as preferred. We also provided two kinds of counting voice (female and male) during exercise. Visitors could observe their movement and download the data and video after they finished the exercise. The exercise score was given by each squat exercise. Readers can find the system at: Biosignal Art.

### 2.2. Motion Analysis and Performance Feedback

Body-weight squats are known to benefit lower body strength and power. Such exercise can improve physical function in the elderly and enhance aerobic capacity and physical fitness (16–18). The recommended frequency of squat is 1–2 sets of 10 repetitions for the beginner and 2–3 sets of 20 repetitions for the advanced (4, 19). We set a 10-repetition squat exercise as one set in this system, and visitors could repeat this exercise as they liked.

In order to measure the movement kinematics during squat exercise, we applied PoseNet, a pose estimation program developed by Google (20). Briefly, PoseNet is a pose estimation program referring to computer version techniques. After feeding through RGB images or video to a convolutional neural network and processed by a special multi-pose decoding algorithm, PoseNet can return the human pose. The pose result contains 2-D x and y coordinates of 17 keypoints, including eyes, nose, shoulders, hips, knees, ankles, and so on. All motion analysis is performed online. Details and computer programs can be found in (21).

**Figure 3:**
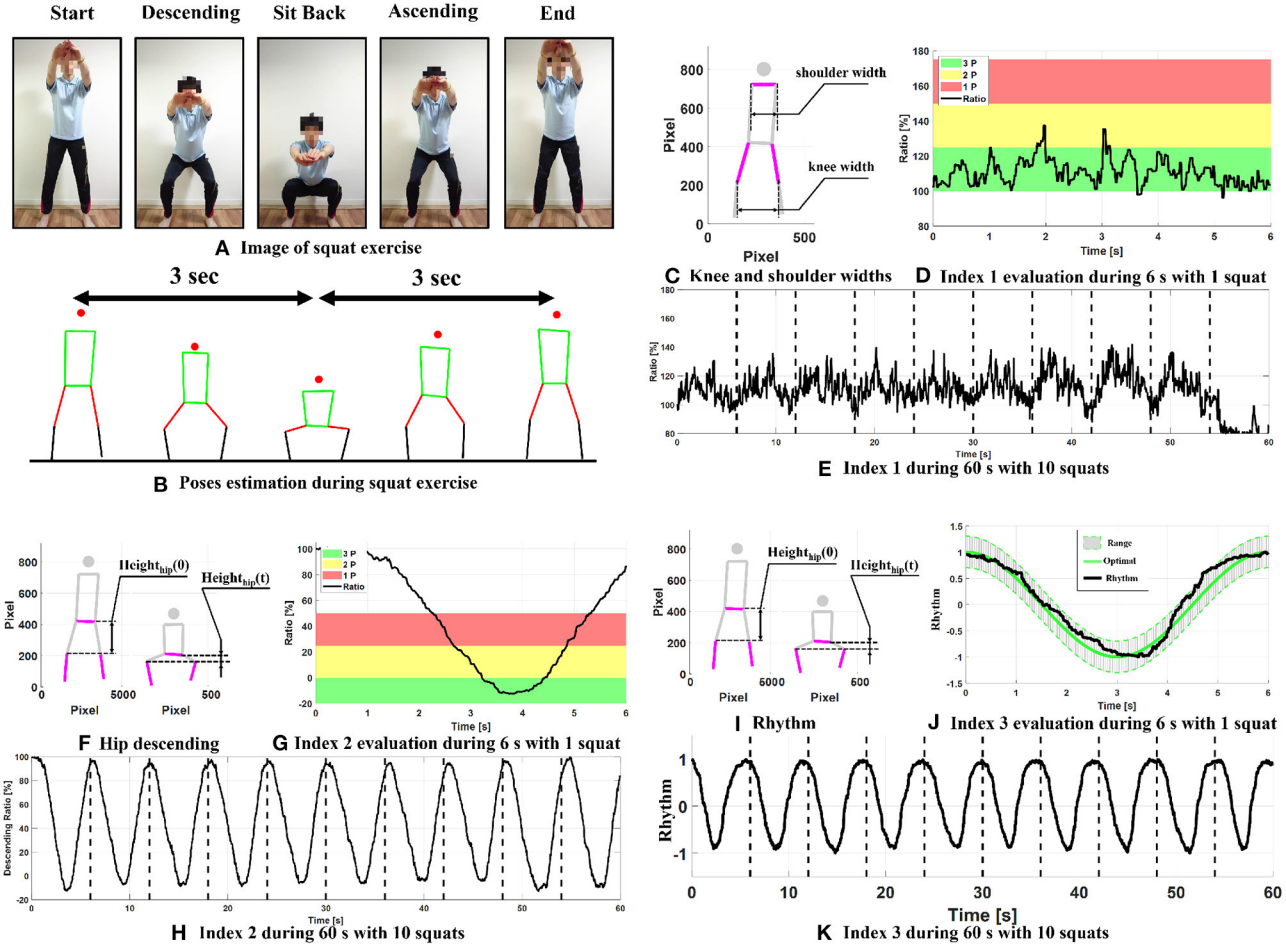
Squat indices. Panels **(A,B)** show the image and pose estimation during squat exercise; panels **(C–E)** indicate Index 1; panels **(F–H)** indicate Index 2; panels **(I–K)** indicate Index 3.

Figures 3A,B show an example of body-weight squat and pose estimation using PoseNet. We defined one squat repetition round as 6 s; thus, one set of the exercise will be 60 s. In every repetition round, first, the exerciser is told to look straight ahead, put their arms parallel to the ground, make sure their knees are not inward; then “sit back” in 3 s, not round the back and bend the knees; at last, lift up in the rest 3 s, instead of standing up immediately. Accordingly, every repetition was to be evaluated according to three indices: knee width, hip position, and squat rhythm, referenced to (22). Higher scores represent the optimal squat exercise.

Figures 3C–E show the definition and evaluation for Index 1: knee width. The ratio between the knee and shoulder widths (Figure 3C) during squat can have a significant effect on the squat kinetics. To minimize the shear force during squat, we expect knee width to be slightly greater than the shoulder width. Accordingly, we set four levels to evaluate knee width:


(1) $$\begin{array}{r} \begin{array}{r} Index\;1=\frac{Width_{Knee}}{Width_{Shoulder}}\times 100\%=\\ \{\begin{array}{cc} 3\;points, & 100\%\leq Index\;1<125\%\\ 2\;points, & 125\%\leq Index\;1<150\%\\ 1\;points, & 150\%\leq Index\;1<175\%\\ 0\;points, & Index\;1<100\%\;or\;Index\;1\geq 175\% \end{array} \end{array} \end{array}$$

Figure 3D shows an example of one squat round. The most frequent point was used for evaluation. In this example, most times during the squat, the ratio was between 100 and 125%, thus, the score in this round is 3 points. Index 1 in one squat set with 10 repetitions is shown in Figure 3E.

Figures 3F–H show the definition and evaluation for Index 2: hip position. Proper hip descending can maximize adaption and minimize the risk of injury. Several types of body-weight squats are recommended for exercisers. Partial, parallel, and full depths are frequently used when evaluating squat (23). Consequently, we set three levels for scoring the squat as follows:


(2) $$\begin{array}{l} \begin{array}{l} \;Index\;2=min\left(\frac{Height_{Hip}\left(t\right)}{Height_{Hip}\left(0\right)}\times 100\%\right)=\\ \;\{\begin{array}{cc} 3\;points, & Index\;2\leq 0\\ 2\;points, & 0<Index\;2\leq 25\%\\ 1\;points, & 25\%<Index\;2\leq 50\%\\ 0\;points, & Index\;2>50\% \end{array} \end{array} \end{array}$$

where *Height*_*Hip*_(*t*) represents the hip height during the squat, *Height*_*Hip*_(0) is the initial hip height. Figure 3G shows the ratio of hip descending during one round. The minimum value of the ratio of hip descending was used as the squat score. In this example, the hip descends to about −10%, which represents a full depth squat; the score for Index 2 in this round is 3 points. Figure 3H shows an example of the hip descending ratio for one squat set with 10 repetition rounds.

Figures 3I–K show the definition and evaluation for Index 3: Rhythm. As aforementioned, we expect one squat exercise to be finished in 6 s with equal rhythm, for both descending and ascending. Thus, the squat rhythm is defined as follows:


(3) $$\begin{array}{l} Rhythm=\frac{2\times \left(Height_{Hip}\left(t\right)\right)-Height_{Hip}\left(min\right)}{Height_{Hip}\left(max\right)-Height_{Hip}\left(min\right)}-1 \end{array}$$

where *Height*_*Hip*_(*t*) is the hip height, *Height*_*Hip*_(*min*) and *Height*_*Hip*_(*max*) are the minimum and maximum hip height, respectively. Squat rhythm score is defined as:


(4) $$\begin{array}{l} \begin{array}{r} Index\;3=\frac{N_{Rhythm}\in \left[-30\%Op,+30\%Op\right]}{N_{All}}\times 100\%=\\ \{\begin{array}{cc} 4\;points, & 80\%⩽Index\;3\\ 3\;points, & 60\%⩽Index\;3<80\%\\ 2\;points, & 40\%⩽Index\;3<60\%\\ 1\;points, & 20\%⩽Index\;3<40\%\\ 0\;points, & 0\%⩽Index\;3<20\% \end{array} \end{array} \end{array}$$

*N*_*Rhythm*_ represents the data point when the squat rhythm is located in the optimal rhythm range, and *N*_*All*_ is the total number of data. Optimal rhythm range (gray area in Figure 3J) was set at ±30% of optimal rhythm curve (green line in Figure 3J), which can be defined by using (5).


(5) $$\begin{array}{l} Optimal=\cos\left(\pi t/3\right) \end{array}$$

In this example, over 80% of points are located between the optimal range; thus, the score for Index 3 is 4 points. Figure 3K shows an example of squat rhythm for one squat set with 10 repetition rounds.

### 2.3. Music Interactive Feed Back

After evaluating the squat performance, a video with music interactive feedback is provided to the visitor for squat reviewing and download. We invited a composer to create four pieces of original background music (BGM) and audio noise for our system. Visitors can choose one type of music during the squat exercise.

**Figure 4:**
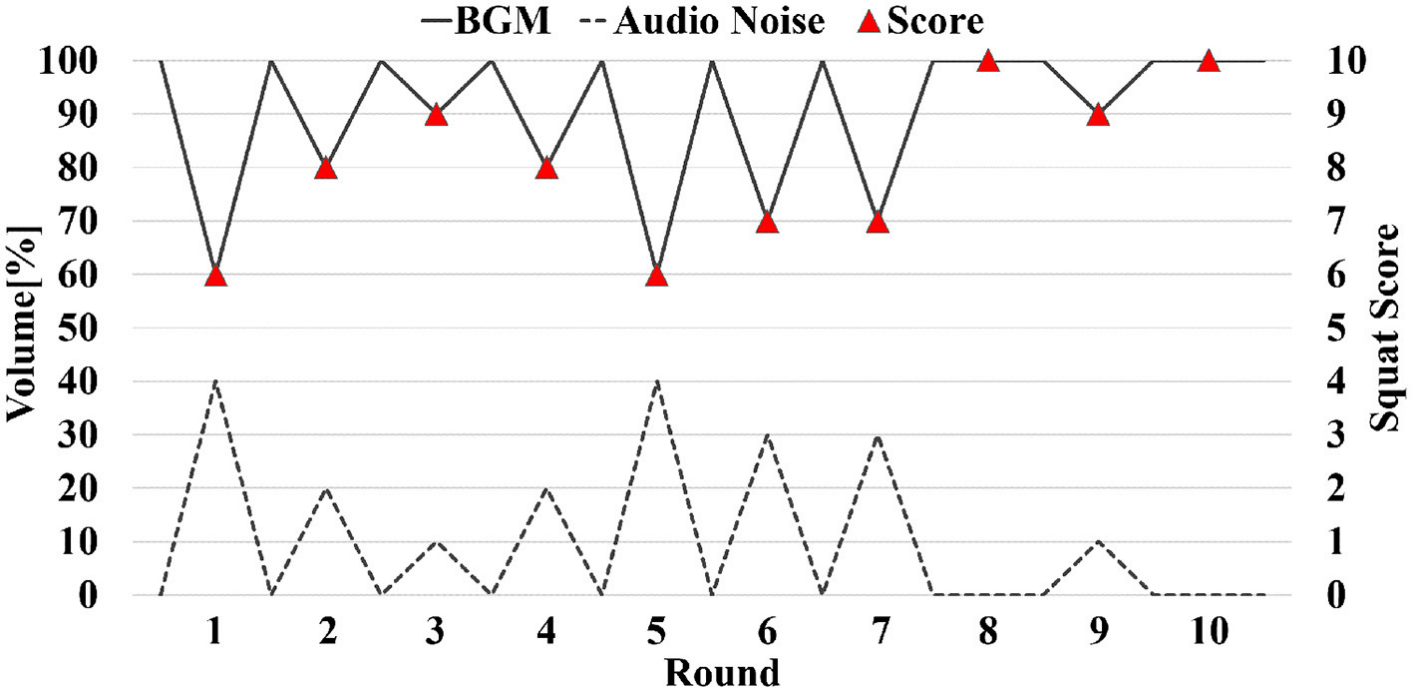
Image of interactive music feedback. The X-axis indicates the squat round, left vertical axis is the volume of BGM and audio noise, and the right vertical axis is the squat score.

Figure 4 shows an example of interactive music feedback. BGM and audio noise are embedded in the video simultaneously, and the volume is adjusted through the squat score. Visitors get clearer music feedback (higher volume for BGM and lower volume for audio noise) when the squat score is high, and vice versa. For example, at round 4, the squat score was 8 points (red triangle), volume of BGM (solid line) was, therefore, set as 80% of maximum, volume of audio noise (dash line) was set as 20% of maximum.

## 3. System Evaluation

### 3.1. Participants

This online fitness system was first released on May 13, 2020, and broadcast through television news, university homepages, and social media platforms such as YouTube, Facebook, and Twitter. Anyone aware of the web link could visit and utilize our system. For this study, we collected data 5 months after the system was released. Only data with complete basic information and web survey answers were used for further analysis.

### 3.2. Web Survey Design

We also designed a self-reported web survey to evaluate the developed system. The survey contents are summarized as follows:

- Basic Information: age, gender;
- Exercise change due to the COVID-19 pandemic: increased, not changed, decreased;
- Exercise Report: Exercise frequency per week (over 5 times/week, 3–4 times/week, 1–2 times/week, 1–2 times/month) and Exercise time (over 60 min/time, 30–50 min/time, 10–20 min/time, less than 5 min/time).

Visitors were divided into three groups according to their age: younger (under 39 years), middle (40–59 years), and older (over 60 years) (24). A PA of 240 min per month is recommended according to the Japanese Ministry of Health, Labor and Welfare (25); thus, we calculated visitors' PA every month by using the data reported in the Exercise Report. Exercise habit is defined as insufficient exercise (less than 240 min per month) and regular exercise (over 240 min per month).

### 3.3. Statistical Analysis

Statistical analyses were performed using JASP (version 0.14.0.0). The normality of data was tested using the Shapiro-Wilk test, and the appropriate tests were applied, depending on whether the data were normally distributed or not. The total squat score among the three groups was analyzed using an independent one-way ANOVA. Effect size (ω^2^) was calculated (an effect size of ω^2^ = 0.14 indicates a large effect, ω^2^ = 0.06 indicates a medium effect, ω^2^ = 0.01 indicates a small effect, and ω^2^ < 0.01 indicates a trivial effect). *Post-hoc* testing was performed if there was a significant difference. The Kruskal-Wallis test was used to analyze differences in each squat index among the three groups. Dunn's *post-hoc* test was performed if there was a significant difference. The difference in squat score among the exercise rounds for each group was tested using Friedman's RMANOVA. Connor's *post-hoc* pairwise comparisons were performed if there was a significant difference in the squat score in each exercise round. Considering that the exercise habit, i.e., whether one has regular exercise habits or insufficient exercise may influence the results of squat performance, it is necessary to confirm the effect of this covariate and control (or remove) the effect if necessary. Thus, a one-way analysis of covariance (ANCOVA) was performed to reveal the relationship between squat scores (each sub-index and total scores), exercise changes, and exercise habits for the three groups. The significance level was set as 5%.

## 4. Results

### 4.1. Data Collection Overview

**Figure 5:**
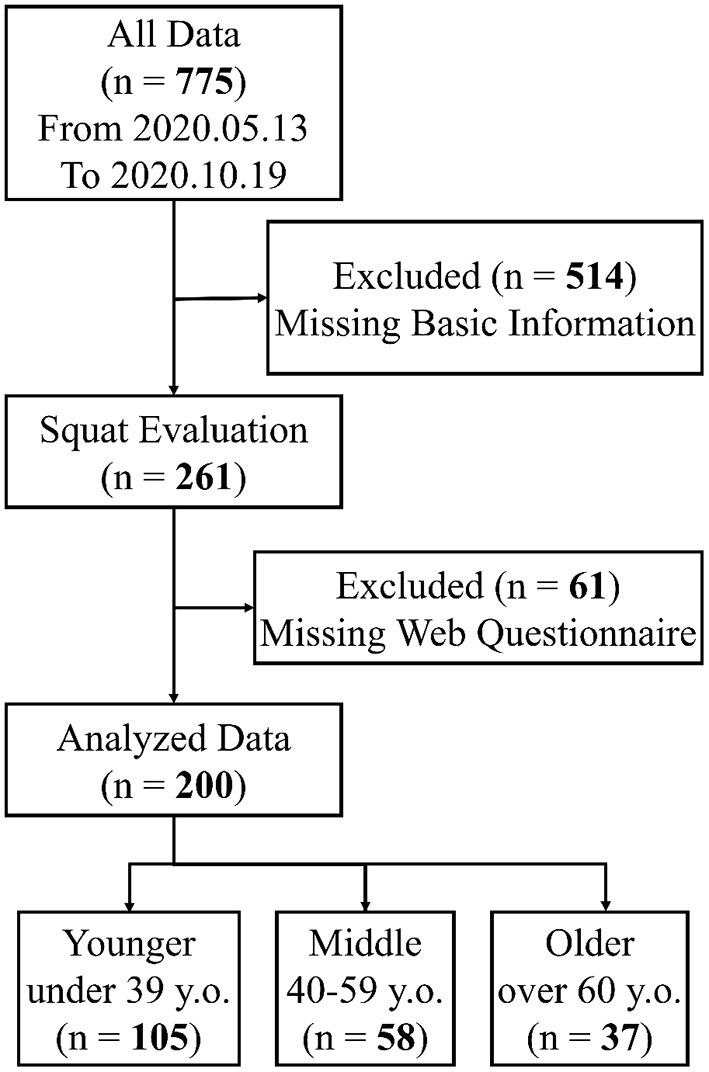
Flow chart of data collection.

Figure 5 shows the flow chart of the data collection. In this study, we collected data for 5 months after the system's initial release, i.e., from May 13, 2020 to October 19, 2020. Data from 775 visitors were screened. Data of 200 visitors who had completed basic information and the questionnaire were divided into three groups, based on their age: younger, middle, and older.

Table 1 shows the characteristics of the three groups. In total, 51.5% of the visitors were men and 48.5% were women. In the analyzed data pool, the number of women visitors were less than that of men in the younger (44 and 61) and older (17 and 20) groups; however, more middle-age women (*n* = 36) used the fitness system than men (*n* = 22).

**Table 1:** Characteristics of the three age-based groups.

**Characteristic**	**Younger**		**Middle**		**Older**		**All**	
	**(*****n*** **=** **105)**		**(*****n*** **=** **58)**		**(*****n*** **=** **37)**		**(*****N*** **=** **200)**	
Age (years)	25.0 ± 6.6		47.6 ± 5.1		75.4 ± 5.7		40.9 ± 20.1	
	(16, 39)		(40, 56)		(60, 87)		(16, 87)	
Height (cm)	165.8 ± 9.6		165.9 ± 7.6		157.8 ± 8.2		164.4 ± 9.3	
	(148.0, 189.0)		(150.0, 179.0)		(145.0, 173.0)		(145.0, 189.0)	
Weight (kg)	60.8 ± 13.3		61.3 ± 10.4		59.2 ± 10.7		60.6 ± 12.0	
	(44.0, 100.0)		(45.0, 88.0)		(40.0, 75.0)		(40.0, 100.0)	
	***n***	**%**	***n***	**%**	***n***	**%**	***n***	**%**
**Gender**								
Men	61	58.1	22	37.9	20	54.1	103	51.5
Women	44	41.9	36	62.1	17	45.9	97	48.5
**Exercise**								
Increased	20	19.0	14	24.1	8	21.6	42	21.0
Not Change	45	42.9	21	36.2	16	43.2	82	41.0
Decreased	40	38.1	23	39.7	13	35.2	76	38.0
**Habit**								
Insufficient	52	49.5	34	58.6	12	32.4	98	49.0
Regular	53	50.5	24	41.4	25	67.6	102	51.0

*Data for age, height, weight: mean ± SD (minimum, maximum)*.

According to the questionnaire results, 38% of visitors claimed decreased exercise due to the COVID-19 pandemic. Compared with the younger and the older groups (38.1 and 35.2%), the percentage of decreased exercise was higher in middle-age group (39.7%). The percentage of visitors who reported increased exercise in the middle-aged group (24.1%) was also higher than that in the other two groups (i.e., 19.0% for the younger group and 21.6% for the older group).

Exercise habit results showed different trends among the three groups. In the younger group, the number of system visitors with regular exercise habits was almost the same as that with insufficient exercise (50.5 and 49.5%, respectively). Only 41.4% of middle-aged visitors reported they had regular physical exercise, but the number of visitors with regular exercise habits in the older group (67.6%) was twice as large as those who did not have enough physical exercise (32.4%).

### 4.2. Squat Performance

**Figure 6:**
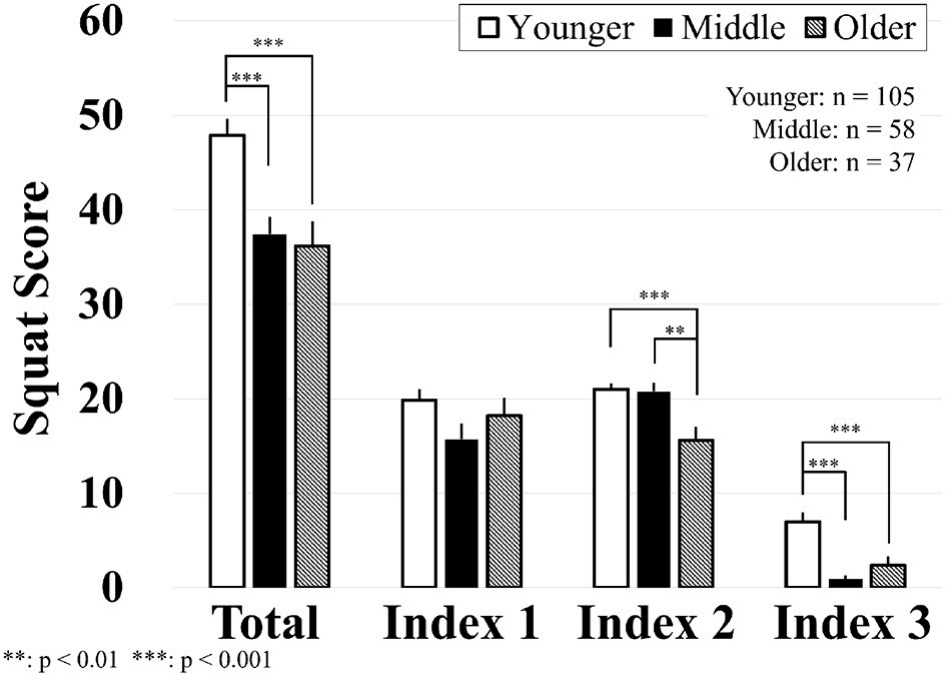
Results of squat scores. Data are mean value and standard error.

Figure 6 shows the results of squat scores. An independent one-way ANOVA showed a significant effect of age on total squat scores *F*_(2, 197)_ = 3018.048, *p* < 0.001, ω^2^ = 0.095. *Post-hoc* testing using Tukey's correction revealed that the younger group had significantly higher scores than the middle (*p* < 0.001) and older groups (*p* < 0.001). There was no significant difference in the squat scores between the middle and old groups (*p* = 0.926).

The Kruskal-Wallis test showed that there was no significant difference in Index 1 among younger, middle, and older groups H(2) = 5.426, *p* = 0.066. Indices 2 and 3 were significantly affected by age, H(2) = 13.069, *p* = 0.001, and H(2) = 24.950, *p* < 0.001, respectively. Dunn's *post-hoc* comparisons showed that Index 2 for the old group was significantly lower than both the younger and middle groups (*p* < 0.001 and *p* = 0.001, respectively). There was no significant difference between the younger and middle groups (*p* = 0.409). Dunn's *post-hoc* comparisons showed that Index 3 for the young group was significantly higher than in both the middle and older groups (*p* < 0.001). There was no significant difference between the middle and older groups (*p* = 0.232).

**Figure 7:**
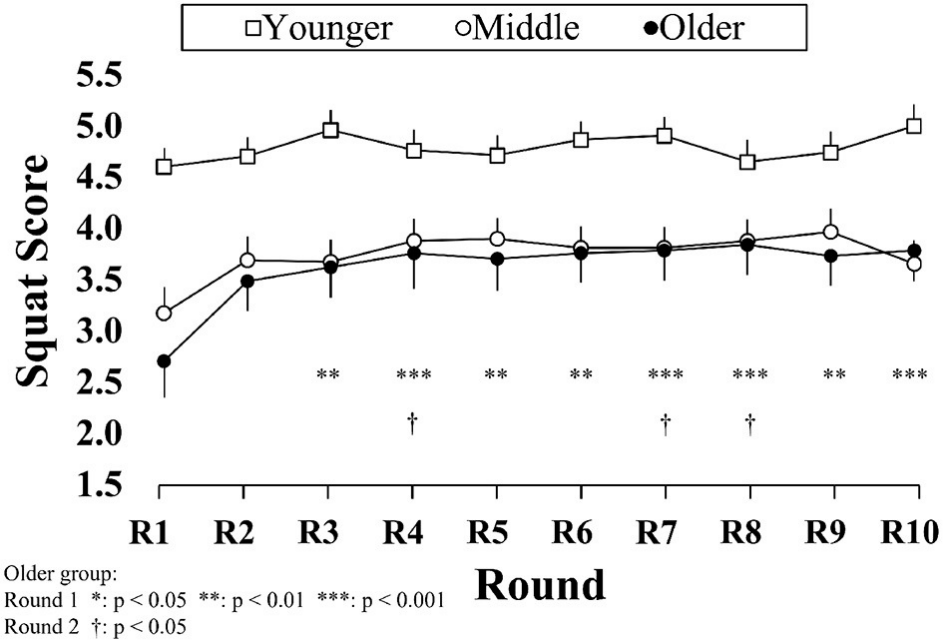
Results of squat scores at each round. Data are mean value and standard error.

Figure 7 shows the results of the squat scores at each exercise round. Friedman's test showed that there was no significant difference in each exercise round for the younger group [χ^2^_(9)_ = 16.109, *p* = 0.065], and middle group [χ^2^_(9)_ = 16.781, *p* = 0.052]. However, the exercise round had a significant effect on squat scores for the older group, χ^2^_(9)_ = 23.535, *p* = 0.005. Conover's *post-hoc* comparisons showed that the squat score in the first round was significantly different from the other rounds (all *p* < 0.05) except for round 2; squat score at the second round was different with rounds 4, 7, and 8 (*p* < 0.05).

### 4.3. Effect of the COVID-19 on Squat Performance

**Figure 8:**
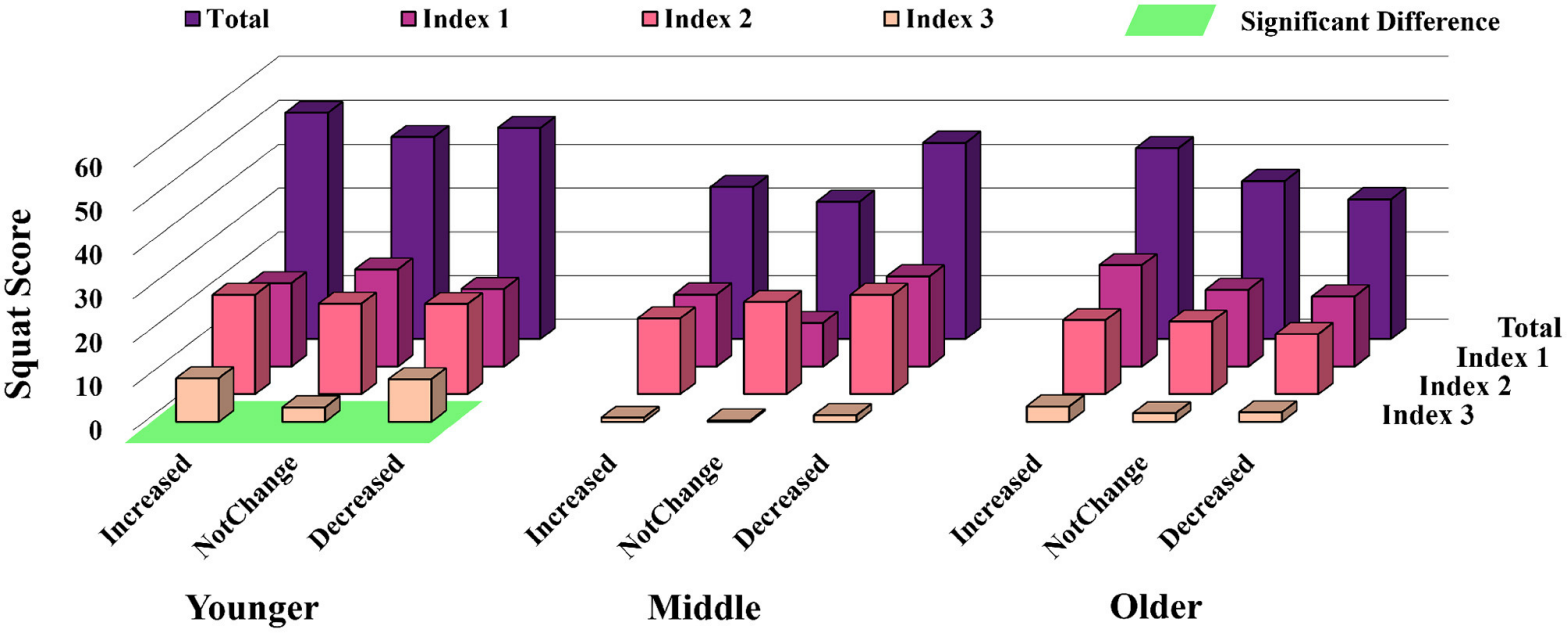
Exercise changed and squat score among different population.

Figure 8 shows the results of the squat scores (each index and the total score) under three situations of changed exercise for the three groups.

For the total squat scores, squat scores for the younger group under the three situations did not change dramatically. However, there was a trend that, among the older visitors, all squat scores dropped when exercise decreased. For the middle-aged group, it was interesting to notice that the squat score for those who reported decreased exercise during the COVID-19 pandemic seemed higher compared to those who had increased and for those who had not changed exercise. Similar to the results in Figure 6, for all groups, squat scores for Index 3 were lower than those for the other two indices in all situations of exercise change. Detailed results of the statistical analysis are shown in Table 2.

**Table 2:** ANCOVA results of squat scores, exercise changed, and exercise habit of three groups.

**Group**	**Index**	**Situation**	**Sum of squares**	**df**	**Mean square**	***F***	***p***	**ω^2^**
Younger	Total	Exercise changed	473.228	2	236.614	0.769	0.466	0.000
		Exercise habit	91.547	1	91.547	0.298	0.587	0.000
		Residuals	31061.422	101	307.539			
	1	Exercise changed	511.483	2	255.741	1.798	0.171	0.015
		Exercise habit	102.722	1	102.722	0.722	0.391	0.000
		Residuals	14361.964	101	142.198			
	2	Exercise changed	68.920	2	34.460	0.779	0.462	0.000
		Exercise habit	0.842	1	0.842	0.019	0.891	0.000
		Residuals	4468.061	101	44.238			
	3	Exercise changed	1296.474	2	648.237	6.922	0.002^**^	0.099
		Exercise habit	375.532	1	375.532	4.010	0.048^*^	0.025
		Residuals	9458.312	101	93.647			
Middle	Total	Exercise changed	659.516	2	329.758	2.183	0.123	0.037
		Exercise habit	591.735	1	591.735	3.917	0.053	0.046
		Residuals	8158.200	54	151.078			
	1	Exercise changed	569.529	2	284.764	2.052	0.138	0.035
		Exercise habit	221.633	1	221.633	1.597	0.212	0.010
		Residuals	7494.400	54	138.785			
	2	Exercise changed	181.890	2	90.945	1.921	0.156	0.031
		Exercise habit	27.741	1	27.741	0.586	0.447	0.000
		Residuals	2556.333	54	47.340			
	3	Exercise changed	2.281	2	1.140	0.162	0.851	0.000
		Exercise habit	17.399	1	17.399	2.473	0.122	0.025
		Residuals	379.935	54	7.036			
Older	Total	Exercise changed	551.960	2	275.980	1.066	0.356	0.004
		Exercise habit	12.496	1	12.496	0.048	0.827	0.000
		Residuals	8541.196	33	258.824			
	1	Exercise changed	195.727	2	97.864	0.701	0.503	0.000
		Exercise habit	36.592	1	36.592	0.262	0.612	0.000
		Residuals	4604.283	33	139.524			
	2	Exercise changed	79.490	2	39.745	0.499	0.612	0.000
		Exercise habit	5.115	1	5.115	0.064	0.802	0.000
		Residuals	2630.467	33	79.711			
	3	Exercise changed	13.684	2	6.842	0.175	0.840	0.000
		Exercise habit	0.064	1	0.064	0.002	0.968	0.000
		Residuals	1292.566	33	39.169			

* *p < 0.05*,

** *p < 0.01. Bold values are indicate significant difference*.

Firstly, for the total squat score, the covariate exercise habit, was not related to the squat score for the younger or older group. The effect of exercise habit on squat performance was close to significance for the middle-aged group [*F*_(1, 54)_ = 3.917, *p* = 0.053, ω^2^ = 0.046]. As a result, there was no significant effect of changed exercise on all squat scores for any of the three groups (all *p* > 0.05). Second, there was no significant effect of changed exercise or exercise habit on indices 1 and 2 for all groups (all *p* > 0.05).

**Figure 9:**
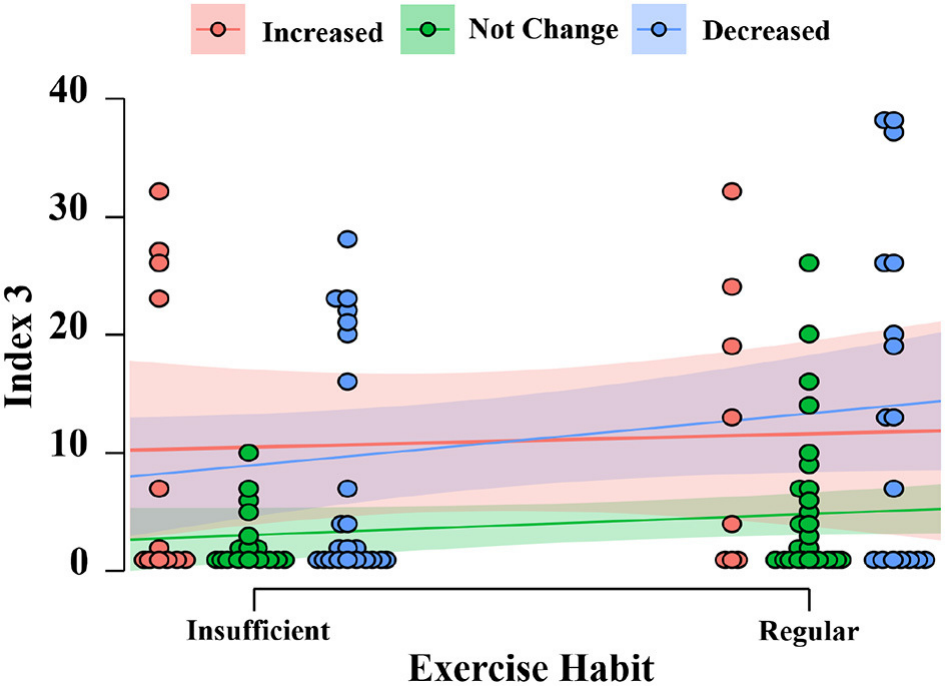
Index 3 for young group. Plots are drawn using mean values and 95% confidence intervals.

However, for the younger group, exercise habit was related to the squat rhythm (Index 3), *F*_(1, 101)_ = 4.010, *p* < 0.05, ω^2^ = 0.025, and it was a small effect. There was also a significant and medium effect of changed exercise on squat rhythm, *F*_(2, 101)_ = 6.922, *p* < 0.01, ω^2^ = 0.099; no significant effect was observed for middle and older groups. *Post-hoc* testing using Tukey's correction revealed that not changing exercise resulted in a significantly lower point of squat rhythm, compared to increased (*p* = 0.016) and decreased (*p* < 0.01) exercise. This can be seen from the plot in Figure 9. There was no significant difference in the squat rhythm between the increased and decreased situation in the younger group (*p* = 0.933).

## 5. Discussion: Lessons Learned

The purposes of this study were: to develop a free-of-cost online fitness system: “Biosignal Art” and then evaluate this system by investigating visitors' squat exercise performance and its relation between the changes in exercise due to the COVID-19 pandemic. In general, the developed system seems usable for the different populations from teenagers to octogenarians. Different squat performances were observed among the three age groups. Meanwhile, the relationship between squat performance and self-reported exercise changed due to the COVID-19 pandemic. To the best of our knowledge, this was the first study to explore primary data about PA during the early COVID-19 pandemic in different age groups in Japan.

### 5.1. System Design

Compared to other fitness systems or apps, whose prices range from $96 to $288 per year (26–29), we believe that a free fitness system is able to benefit more populations, especially in the face of the worst recession of the global economy (30, 31). Given that the development and delivery of free online fitness services through non-profit or government entities were expected to be one of the solutions to health problems during the COVID-19 pandemic (13), the most significant contribution of the developed fitness system is that “Biosignal Art” is totally free, and provides a new online fitness option. Additionally, the developed system requires no accessory equipment, such as Kinect©Microsoft or Fitbit smart watch©Fitbit, but only a web-camera either built-in or an add-on, which could minimize the users' economic burden.

Body-weight squat has been considered as a valuable primary PA and an optimal home-based exercise (32, 33). Like other physical activities, inappropriate squat performance may impair exercise efficiency and even result in a higher risk of injury (34). There are several points that need particular attention during squat exercises. Normally, there are three kinds of knee widths for squat: wide, narrow, and shoulder widths. A wide knee width was found to increase the patellofemoral and tibiofemoral compressive forces in the knee joint and a narrow width increases forward knee translation and heightens anterior shear forces (35). Therefore, a moderate stance with shoulder width or slightly wider was recommended in our system. As the largest muscle in the hip, the gluteus maximums extend the femur and stabilize the pelvis during the squat. Without proper recruitment of the gluteus muscles, other muscles have to bear the load of the squat. This is suboptimal and may result in muscle imbalance (35, 36). Inadequate depth cannot ensure the training effect, whereas sitting back too deep with difficulty may put users in a dangerous situation such as losing postural balance and falling, especially for older people. Thus, referring to (23), we set three levels of evaluation for the hip position.

Rhythm, also known as tempo, is another essential factor during physical activity. Descending too fast, “drop into the apex of the descent,” can be dangerous when muscles are forced to stretch too much and too fast (34). As a consequence, we expected exercisers to descend and ascend with equal tempo in 6 s.

Previous studies have shown that music during or before PA can offer potential benefits to exercise performance, enjoyment, and physiological efficiency (37–39). However, to the best of our knowledge, most of the fitness systems only provided some BGMs; further, there was no interactive music feedback so far. In our system, not only the visual (video and scores), but also the audio feedback (interactive music that was made by the visitor through his or her own squat performance) was provided. Some visitors reported that the feedback of the squat performance with music was interesting, and interactive music made it clearer and easier when they were setting their fitness goal, and not only according to the scores. Thus, it is considered that the interactive music provided in our fitness system, which consists of originally composed BGM and audio noise, could provide an extrinsic factor of exercise motivation.

As shown in Figure 2, in general, we utilized videos and illustrations to introduce our system and some key points during exercise. In order to make it easier for visitors to operate, buttons related to the fitness process were highlighted using a warm color, whereas the background was designed using a dark color. To date (December 10, 2020), we did not receive any report from the visitors about the difficulty in using our system. It seems that “Biosignal Art” is accepted as a user-friendly interface system.

There are still some drawbacks that need to be improved in order to improve user experience. In the 1st release version of “Biosignal Art” (May 13, 2020), only squat was available. From the users' feedback, we noticed some requirements for system updates to more physical activities for the upper limbs. In response, we added another exercise: body weight shoulder press, in early July in the second system version. We aim to enrich the exercise categories in the next published version. Currently, the system language is Japanese only; therefore, it is urgent to provide other language options in the future.

During our initial development stage, we considered that real-time feedback about exercise performance would be very useful for exercisers. However, it was difficult to use only the web-based service because of the massive postural estimate calculation. On the other hand, providing a music interactive video requires a faster transmission speed of the network. With the increased complexity of software and popularization of 5G technology, it is possible for real-time feedback by developing software or apps.

We used a cloud storage for uploading and saving the data in our system, except for the video. The video can only be downloaded by the visitor and we did not save any individual's video in our system. As data that contains any information which may identify any individual person were not used in this system, it was almost impossible to record the exercise history for a particular visitor. Although visitors' privacy could be well-protected through anonymization, further studies investigating training efficiency within exercisers turned out to be difficult because we could not track visitors' exercise performance. By providing free software for a PC and an app for a smartphone with a user registration system, we can easily perform a follow-up study on fitness improvement.

In our study, we initially considered providing a multi-exerciser mode for the visitors who may be willing to share and join in our system with their friends and families. However, we decided to provide only a single-exerciser mode, owing to technical limitations, such as the camera set-up and accuracy of motion estimation. It is noteworthy that older people who live alone may become lonelier and more depressed during the COVID-19 pandemic (40). Moreover, loneliness and social isolation are not only linked to worse physical health, but also to higher mortality (41, 42). Working out with their friends, families, or even strangers may relieve the lonesome and arouse the motivation of PA. In future work, we aim to improve our system in order to support multi-exercisers mode.

### 5.2. System Setup

The setup of “Biosignal Art” is simple and age-appropriate, especially for older users. Users need a PC (Windows or Mac, with built-in web camera) or smartphone (for Android only). There is no need to install other software or download any app, and users do not have to register to access the system.

To capture the full body, the camera has to be set up at least 1.5 m in front of the visitors. Here, two problems arise. First, some visitors reported that they had difficulty in fitting their full bodies into the camera. Different types of homes, furniture arrangements, and available space in front of the computer may make it difficult to set the camera. Using a wide-angle camera could solve this problem without having the need to rearrange the furniture or the computer to accommodate the system. The second problem is that it may be difficult to see the screen during the squat exercise. The initial test of our system was performed at the laboratory using a monitoring screen of 24 inches. A computer with a larger monitor or contact with the TV screen is recommended.

### 5.3. Motion Tracking

Motion tracking in our system relies on the PoseNet pose estimation program. PoseNet is a neural network that can return real-time human pose estimation and has been widely used for web app development as an open source program (43). The most significant advantage of this method is that there is no need to prepare other equipment for motion tracking, which can simplify the system setup and release the user's burden.

There are 17 different body points that PoseNet can predefine, including the nose, eyes, ears, shoulders, elbows, wrists, hips, knees, and ankles. As shown in Figure 3, only nine points were used for squat evaluation in this study. If we can track the necessary body parts only, it is possible to improve the pose estimation speed, and thus real-time feedback could be provided to our visitors.

At the present stage, we only evaluated squat performance from the front view. Considering that some evaluation indices such as the hip position would be much easier for exercisers' self-monitoring, side-view estimation is necessary. Here, it is necessary to point out that tracking squat motion from both front and side views simultaneously may require additional equipment and more space.

Owing to the algorithm of PoseNet (computing heat maps and offset vectors) (43), we can only acquire the relative positions of the key points in the captured image or video. It is difficult to calculate the precise distance without calibration. However, as the motion analysis mainly relies on the relative position, PoseNet could meet our needs.

### 5.4. Squat Evaluation

It was found that the total squat score for the younger group was significantly higher than the middle and the older groups, but we surprisingly found that there was no difference between the middle and older aged participants. Squat performance has been studied between the younger and the older population, and squat is known as an appropriate rehabilitation exercise for post-ligament reconstruction surgery (44, 45). To the best of our knowledge, this is the first time that squat exercise has been studied among younger, middle-aged, and older populations in Japan. Moreover, knowledge about squat performance for middle-age populations is still unknown. Compared to the upper limb, a dramatic decrease in muscle mass at the lower extremity was observed in Japanese people after their 20s (46). Decreased muscular power seems to result in a lower squat score for older people. Moreover, time spent sitting (sedentary time) as well as insufficient PA, is reported to increase the risk for functional decline and muscle mass loss (47). According to the results from (48), the proportion of sedentary time for the middle-aged population is higher than that for the old population, both, for men and women, in Japan. Therefore, it is speculated that, although middle-aged people have more muscle power than older people, too much time spent sitting impairs the ability of PA.

As mentioned, a wider knee width may increase the patellofemoral and tibiofemoral compressive forces up to 15% during squat descending, and the inward knee will increase shear forces (35). From the bar chart at Figure 6, scores for Index 1 of the three groups were about 20 points (average 2 points at each round), indicating that exercisers made an appropriate stance position with their knee slightly wider than their shoulder under each round of squat exercise. The developed fitness system seems to be able to provide optimal stance instructions.

During the squat, the downward distance of the hip for the old group was shorter than it was for both the younger and the middle-age groups, as evidenced by a lower score in Index 2. The score of Index 2 for both younger- and middle-aged groups was 20 points (2 points at each round), indicating that both groups could “sit back” to 25% of the initial hip height. However, the score of the older group was about 15 points; in other words, older people could only descend the hip to less than 50% of the initial hip position. Yuhara et al. reported a similar finding: with aging, hip depth during squat dramatically decreased in older people (49). The shallower squat depth observed in this study indicates a lack of strength and stability of the hip musculature or asymmetrical strength of hips for older people.

It was noticed that squat rhythm scores were the lowest, compared to the other two indices in all three groups. Although the younger-aged group showed the highest score compared to the other groups, controlling the tempo of descending and ascending was the most challenging task during squat exercise. In this study, we expected exercisers to finish one squat in 6 s, with the equal time of descending and ascending and defined the optimal rhythm using a cosine curve. Myer et al. (34) suggested a 2:1 up to 4:1 (descending–ascending) rhythm for squat exercise. Given that poor squat rhythm in older people may indicate a lack of lower limb eccentric strength control and the inability of balance control, it was considered that rhythm variation could be too difficult for older people and non-well-trained exercisers.

It is interesting to note that total squat scores at each exercise round showed a different trend in the three groups (see Figure 7). For the young exercisers, squat scores were maintained at a high level (above 4.5 points) even in the first round of exercise, whereas the score in the first round decreased with aging: 3.1 points for the middle-aged group and 2.7 points for the old-aged group. A significant difference in the squat score for the old group between the first round and the rest rounds was considered to be related to “fear of falling,” related to self-defense mechanisms (49). Fear of falling is known to be associated with poorer PA performance (50, 51). In order to lower the risk of falling, when performing some demanding motion that requires massive muscle strength, such as a squat, older people do not tend to make the full effort because they may not be confident in their muscle power. After several trials, being aware of the low risk of falling, older people would truly start the activity.

In conclusion, the younger-aged group had the best squat performance. All age groups could make an appropriate stance with moderate knee width. The middle-aged group could descend the hip as deep as the young group, whereas older people had a shallower hip position during squat exercise. The squat rhythm seemed the most challenging task during squat, where the middle- and older-aged groups had worse squat tempo than the younger group. Older people had lower scores at the beginning of the squat exercise.

Some visitors gave the feedback that they could not totally understand the importance of each index and some others required more evaluated indices. Thus, in future work, we will consider adding more specific introduction and guidance, such as breath method and head and foot position during squat exercise.

### 5.5. Social Aspect: Physical Activities During the COVID-19 Pandemic

Increased time of sitting and muscle disuse due to the COVID-19 pandemic may result in decreased muscle power, strength, and function (52). Thus, it is more important for people to keep in health. However, closed gymnasiums and other public sports facilities limited people's ability to engage in enough physical activities. According to the results of a social survey conducted on May 15, 2020, from one thousand Japanese citizens (53), about 18% of respondents decreased indoor activity, and 25% decreased outdoor PA. In this paper, we reported more cases of decreased PA decreased; 38% of visitors reported decreased PA time due to the COVID-19 pandemic.

Meanwhile, we found that 49% of our system visitors did not exercise enough. It was noticed that even though people reported that they did not get enough exercise, 66% of them still could not increase PA (53). Lack of access and training skills may deteriorate this situation during the COVID-19 pandemic. The “Biosignal Art” system is expected to become a new option for online fitness system.

As showed in Figure 8, squat scores for each index differed with the change in exercise in different aged populations. It is necessary to point out that specific discussions about the relationship between squat performance and changed exercise is beyond the scope of this paper. Meanwhile, we did not collect any detailed information about self-reported exercise, such as the category or the calorie consumption of the exercise. Thus, it was difficult to have an in-depth discussion of these points. In this paper, we only present some general discussions from the short-term results.

For the younger-aged group, compared to those with increased exercise, there was only a slight decrease in all squat scores among those who reported no change and decreased exercise. It was considered that even though they could not have the same exercise as before the COVID-19 pandemic, young exercisers still had enough physical ability to fulfill squats of high quality. Although there was no significant difference in the squat scores in Indices 1 and 2, the young group who reported no change in physical exercise had the lowest squat points at Index 3, regardless of whether they had regular or insufficient exercise so far (see Figure 9). The reason for this is still unknown.

It was surprising to find that, for middle-aged exercisers, the squat score was higher for those who reported decreased exercise. The percentage of people who reported decreased exercise due to the COVID-19 pandemic in the middle-aged group was higher (39.7%) than the younger-aged and older-aged groups (38.1 and 35.2%, respectively). Ding et al. found that the COVID-19 lockdown may have led to an increase in people's interest in PA and improved awareness of physical exercise (54). Therefore, it was speculated that stronger motivation inspired people to make more effort during the squat exercise. For older people, decreased PA time resulted in worse squat performance, both, for sub-indices and the total score. Similar to the finding in (8), our results indicated that decreased physical exercise due to the COVID-19 pandemic had a negative effect on exercise performance for older people in the short-term, and it may become a permanent entrenched risk of health problems.

However, there are some limitations to this study. As “Biosignal Art” is a totally free and open-access, visitors who accessed our system and analyzed in this study cannot be controlled. We collected data from May 13, 2020 (when the state of emergency was extended to all prefectures in Japan) to October 19, 5 months after the first release of our system. A total of 775 data points were collected, and 200 data points were analyzed. Although this was a limited data number, we provided primary data about exercise change due to COVID-19 among different populations. Moreover, the results of this study indicated the influence of COVID-19 on particular exercise performance for different ages in Japan, for the first time, to the best of our knowledge. But it is necessary to emphasize that finding in this study can only be generalized for Japan's population, because the present system language is Japanese only, and all system visitors came from Japanese domestic. Although there was no significant difference in the short-term change, a decreased trend of squat scores in older people may suggest a decrease in lower limb physical function and disability in the near future.

In this study, we used a self-reported web survey to investigate visitors' exercise habit. If the visitor's total exercise time was less than 240 min per month, he or she was defined as “insufficient exercise” (25). As aforementioned, the limitation of this survey method was that it lacked detailed exercise category, and the calorie consumption of the exercise was also unknown. Fortunately, use of wearable technology, such as Fitbit band and Apple watch, allows us to easily collect users' physical activity data on a long term (55). But it remains an open aspect when consumer grade wearables are largely used, which is the ethical problems: how to manage such a big amount of individual data and how to solve the related privacy and security issues (56).

## 6. Conclusion

In this paper, we designed a free online squat fitness system: Biosignal Art, and evaluated it by investigating the visitors' squat exercise performance and its relation to the change in exercise due to the COVID-19 pandemic. In general, the developed system seems usable for the different populations from teenagers to octogenarians. Younger people showed better squat exercise performance than middle-aged and older people. With the stance instruction from Biosignal Art system, all age groups successfully performed optimal knee width during squat exercise. Compared to younger people, older people showed shallower squat depth and worse squat rhythm. Although the short-term study did not show significant effect of changed exercise on peoples' squat performance, we observed a decreased trend of squat performance in older people, which may indicate the possibility of a negative effect of the COVID-19 pandemic on older people's exercise performance, and it may become a risk of health problem in the future.

At last, we summarized the implications and future direction according to the lessons that were learned from this study. For the future system design and setup, it is necessary to focus more on multi-language, exercisers, and categories system, thus more people with their friends and family can keep sufficient physical activity. It is also necessary to make a registration system, or link the fitness system to the user's wearable device in order to track visitors' fitness performance, if users' privacy can be well-protected. Faster pose estimation in order to provide real time feedback is expected. Other exercise categories as well as more detailed introduction/guidance, including breath rhythm are urgently needed.

## Data Availability Statement

The raw data supporting the conclusions of this article will be made available by the authors, without undue reservation.

## Ethics Statement

Ethical review and approval was not required for the study on human participants in accordance with the local legislation and institutional requirements. Written informed consent for participation was not provided by the participants' legal guardians/next of kin because All users agreed to the privacy policy and terms of service through the developed system online.

## Author Contributions

TW wrote the first draft of the manuscript. TW and SO contributed to the conception and experiment design. TW, MK, SO, and NS contributed to the construct of Biosignal Art system. SS and SM supported the motion analysis. RO created the original background music. TW and MK collected and managed experiment data. TW, SO, SS, and NS contributed to data analysis, results, and discussion. All authors contributed to manuscript revision, read, and approved the submitted version.

## Conflict of Interest

The authors declare that the research was conducted in the absence of any commercial or financial relationships that could be construed as a potential conflict of interest.
